# Stromal sensitivity to radiation and hyperthermia.

**DOI:** 10.1038/bjc.1987.211

**Published:** 1987-10

**Authors:** S. A. Hill, K. A. Smith, J. Denekamp

**Affiliations:** Cancer Research Campaign, Gray Laboratory, Mount Vernon Hospital, Northwood, Middlesex, UK.

## Abstract

The influence on stroma of heat alone, X-rays alone or the combined treatment, has been studied using the tumour bed effect (TBE) as an assay. Ca NT cells have been implanted into previously treated subcutaneous sites as an angiogenic stimulus. The vascular damage is then assessed by the reduced tumour growth rate, which results from inadequate vascular proliferation. A range of X-ray doses was used and large alterations in latent period for growth to 2 mm diameter were followed by smaller alterations in the growth rate of established tumours. A dose response relationship was seen for latency (0-20 Gy) and for growth rate (0-16 Gy). A range of subcutaneous temperatures was obtained by immersion in a water bath for 60 minutes at 40 degrees, 41.5 degrees, 43 degrees or 44.5 degrees C. A slight retardation of tumour growth was seen after 41.5 degrees C, but an unexpected acceleration resulted from the highest heat treatment. Combined heat and X-ray treatments showed thermal sensitization of the X-ray induced TBE at 41.5 degrees C, with a reversal at higher temperatures. At 43 degrees C and 44.5 degrees C a mild thermal burn was induced and this appeared to elicit neovascularisation that could be utilized by the implanted tumour cells. Delayed implantation of tumour cells (at 4 weeks instead of 1 day) abolished this effect.


*Br. J. Cancer* (1987), **56**, 383–388 © The Macmillan Press Ltd., 1987

### Stromal sensitivity to radiation and hyperthermia

S.A. Hill, K.A. Smith & J. Denekamp

*Cancer Research Campaign, Gray Laboratory, Mount Vernon Hospital, Northwood, Middlesex, UK.*

**Summary** The influence on stroma of heat alone, X-rays alone or the combined treatment, has been studied using the tumour bed effect (TBE) as an assay. Ca NT cells have been implanted into previously treated subcutaneous sites as an angiogenic stimulus. The vascular damage is then assessed by the reduced tumour growth rate, which results from inadequate vascular proliferation.

A range of X-ray doses was used and large alterations in latent period for growth to 2 mm diameter were followed by smaller alterations in the growth rate of established tumours. A dose response relationship was seen for latency (0–20 Gy) and for growth rate (0–16 Gy). A range of subcutaneous temperatures was obtained by immersion in a water bath for 60 minutes at 40°, 41.5°, 43° or 44.5°C. A slight retardation of tumour growth was seen after 41.5°C, but an unexpected acceleration resulted from the highest heat treatment.

Combined heat and X-ray treatments showed thermal sensitization of the X-ray induced TBE at 41.5°C, with a reversal at higher temperatures. At 43°C and 44.5°C a mild thermal burn was induced and this appeared to elicit neovascularisation that could be utilized by the implanted tumour cells. Delayed implantation of tumour cells (at 4 weeks instead of 1 day) abolished this effect.

The response of tumours to radiation is often attributed to direct tumour cell kill (e.g. Barendsen & Broerse, 1969). However, damage to the vasculature may also lead to delayed cell death. Thomlinson and Craddock (1967) observed a second wave of growth delay in irradiated tumours when they had grown to 2–4 X treatment size. They attributed this to progressive vascular failure as the angiogenic stimulus of tumour growth resumed. Thus analysis of tumour growth rates following therapy may allow some estimate of the vascular component of injury to be made. A more direct assessment of stromal damage can be made by pretreating a site *before* tumour implantation. In this case any effects on subsequent tumour growth must be the result of stromal damage since the tumour cells are not subjected to the treatment. Pre-irradiation of implant sites has long been known to produce a slowing of the growth of many (though not all) tumours, this has been termed the Tumour Bed Effect (TBE) (e.g. Frankl & Kimball, 1914; Stenstrom *et al.*, 1955; Hewitt & Blake, 1968; Clifton & Jirtle, 1975; Ito *et al.*, 1985).

While only limited attention has been paid to the vascular component of tumour response to radiation, a great deal of emphasis has been placed on this aspect of the response to hyperthermia. Many authors have shown reductions in blood flow and blood volume after moderate heat treatments to tumours (for reviews see Song, 1984; Emami & Song, 1984; Reinhold *et al.*, 1985; Reinhold & Endrich, 1986). It is generally believed that vascular collapse, which occurs within hours after heating, leads to the death of many dependent tumour cells over the subsequent few days (Marmor *et al.*, 1979; Song *et al.*, 1980; Fajardo *et al.*, 1980; Rofstad & Brustad, 1986). Few studies have been performed with the TBE assay to investigate this effect. It has the difference, of course, that the vasculature is quiescent at the time of treatment, and is only forced into proliferation by the subsequent angiogenic stimulus of the implanted tumour cells. Since stimulated endothelial cells *in vitro* are more thermosensitive than quiescent cells (Fajardo *et al.*, 1985) this model may underestimate thermal damage to vasculature in growing tumours. Nevertheless it does allow the effects of radiation, heat, etc. on the vasculature to be elucidated.

We have investigated the effects of treatment of the subcutaneous stroma with hyperthermia, X-rays, or a combination of the two, on the subsequent growth of implanted tumours. Since these modalities are combined in clinical practice it is important to be able to distinguish the vascular and direct components of damage from each.

Correspondence: S.A. Hill.
Received 9 February 1987; and in revised form, 28 April 1987

### Materials and methods

Female CBA/Ht Gy f BSVS mice (10–16 weeks) were used to investigate the response of the subcutaneous stroma to radiation and hyperthermia. Stromal sensitivity was assayed by implanting Ca NT tumour cells into the centre of a previously treated field on the mouse dorsum and comparing the subsequent growth of the developing tumours with that of tumours growing in untreated sites.

### *Irradiation*

For irradiation, unanaesthetised mice were placed in rectangular lead boxes, designed for irradiation of tumours implanted on the rear dorsum (Sheldon & Hill, 1977). A cut-away at the rear of the jig allowed the intended tumour implant site (which had been previously shaved) to be irradiated with a horizontal X-ray beam. All irradiations were performed using a 250 kV Pantak X-ray set, operating at 240 kV and 15 mA, giving a HVL of 1.3 mm Cu and a dose-rate at the centre of the field of 3.6 Gy min$^{-1}$. Six mice were irradiated simultaneously and turned through 180° halfway through each irradiation, to maximise dose uniformity. Before removal from the jigs, the boundaries of the irradiation field were marked on the mouse skin with ink.

### *Hyperthermia*

Local heating was applied by partial immersion in a temperature-controlled waterbath (±0.05°C) for 60 min. The mice were anaesthetised with 60 mg kg$^{-1}$ sodium pentabarbitone and lightly taped into perspex cradles which were suspended on the surface of the water. A 2 cm diameter hole in each jig allowed the chosen area of dorsal skin to be in direct contact with the circulating water. Temperature measurements performed using micro-thermocouples indicated that the skin was maintained within 0.1°C of the temperature of the surrounding water, while the underlying muscle stabilized approximately 1°C below this. Heat alone was used, or heat given after graded doses of X-rays. Three separate protocols were used, with the interval between the two modalities being 0, 4 or 24 h.

### *Tumour*

The tumour used, Ca NT, arose spontaneously in a CBA

mouse in 1968, and has since been serially passaged in syngeneic mice. It is a poorly-differentiated adenocarcinoma with a volume doubling time of 4 days between 5.0 and 6.3 mm mean diameter (65–130 mm$^3$). Pre-immunization of the mice with heavily irradiated tumour cells does not influence the number of cells required to initiate tumour growth, indicating that there is no evidence of immunogenicity in this system (Begg and Smith unpublished data).

*Assay*

Either one or two days after local irradiation and/or hyperthermia, $2 \times 10^5$ tumour cells were injected s.c. into the centre of the treated area. Once the tumours became palpable they were measured 2 to 3 times per week in three perpendicular diameters, until a geometric mean diameter of 10 mm was exceeded, or until the mice became sick due to the presence of lung metastases. Dose response curves were then constructed by plotting the latent time for tumour appearance (the mean time required for tumours to grow to 2 mm mean diameter) or alternatively the mean growth rate for the group against radiation dose (or temperature). Tumour growth rates were calculated by linear regression analysis of the growth curves for individual tumours at sizes of 5 mm diameter and above.

### Results

*Single treatments*

Figure 1 shows the growth curves for groups of Ca NT tumours implanted into either untreated sites or sites which had been previously treated with a single dose of X-rays, or hyperthermia, before tumour cell inoculation. Preirradiation of the tumour bed (Figure 1a) resulted in a dose-dependent lengthening of the latent period (i.e. the time to reach a mean diameter of 2 mm), followed by a slight slowing of tumour growth (for doses up to 16 Gy). At the highest dose, after a long latency of 60 days, the growth rate was similar to that of tumours growing in untreated sites.

Pretreatment with hyperthermia had a distinct but smaller effect on tumour growth (Figure 1b). Low doses of heat (40 or 41.5°C for 1 h) resulted in a slightly later tumour appearance than tumours implanted into untreated sites. However, when higher temperatures were employed (43 and 44.5°C) tumours appeared *earlier* and, after the highest temperature, even grew somewhat *faster*.

The dose response curves for changes in the rate of growth of established tumours are illustrated in Figure 2. With increasing radiation dose to the stroma (Figure 2a), a progressive reduction in tumour growth rate was measured, reaching a minimum at 16 Gy. After the highest dose of 20 Gy, tumours grew almost as fast as in untreated beds. The equivalent dose-response curve for hyperthermia is shown in Figure 2b. At low temperatures the growth rate changes were minimal but tumours grew significantly *faster* after a 44.5°C treatment to the bed.

The TBE has been shown to be maintained for many months after treatment with radiation (Hewitt & Blake, 1968), with the stromal injury remaining latent until the angiogenic stimulus is applied. To investigate whether the accelerated growth persisted after a high thermal dose, tumour cells were implanted either 24 h or 28 days after heating the stroma for 1 h at 44.5°C. Figure 3 shows that the apparent stimulation of tumour growth after this temperature was lost when tumour implantation was delayed

**Figure 2** Mean tumour growth rate (mm day$^{-1}$) as a function of radiation or heat dose, calculated by least squares linear regression analysis of individual tumour growth data between 5 and 10 mm mean diameter. The hatched region represents the mean growth rate ± 1 s.e. for tumours growing in untreated sites.

**Figure 1** Tumour growth after subcutaneous implantation into sites which 1 or 2 days previously had been treated with either a single dose of X-rays or 1 h of hyperthermia. Errors are ± 1 s.e. for groups of 6 mice.

**Figure 3** Growth curves of the Ca NT after immediate or delayed implantation into control or pretreated sites. *Upper panel*: tumour cells implanted 1 day (open symbols) or 1 month (closed symbols) after a single dose of heat. *Lower panel*: tumour cells implanted 1 day (open symbols) or 6 months (closed symbols) after a single dose of X-rays. Different numbers of cells were injected in the two separate experiments, which account for the different positions of the control curves.

for 1 month, whereas the growth-slowing effect of radiation was still apparent 6 months after treatment (lower panel).

### *Combined treatments*

In order to determine whether hyperthermia would have a sensitizing effect on the radiation response of the stroma, heat treatments were applied immediately after graded doses of X-rays. Figure 4 shows the dose-response curves for X-rays followed immediately by 1 h of hyperthermia at temperatures ranging from 40 to 44.5°C. The hatched area is the response to X-rays alone. Since latency showed a dose-dependency over the complete radiation dose range used, data have been expressed only as the time required to grow to 2 mm mean diameter.

A 40°C treatment to the stroma did not enhance the radiation-induced tumour bed effect. Indeed the only temperature which did enhance the radiation response of the stroma was 41.5°C. At this temperature, the dose-response curve for the combined treatment is shifted to the left. Increasing the temperature to 43°C resulted in the loss of this sensitization, while a 44.5°C treatment completely abolished and even reversed the growth slowing effect of the radiation dose which immediately preceded it. Following implantation into a bed which had been heated for 1 h at 44.5°C, all tumours grew faster than those in untreated beds and took approximately the same length of time to reach 2 mm, regardless of the radiation dose involved.

To investigate whether the thermally induced modification of the TBE could be maintained when the radiation and heat doses were separated in time, the waterbath treatment was applied either 4 h or 24 h after irradiation of the tumour bed. With a 4 h interval (Figure 5), the enhancement of the TBE previously produced by a 41.5°C treatment (Figure 4), is no longer apparent. The dose response curve for the combined treatment at 43°C has also been shifted to the right, indicating a reduced TBE relative to that seen with radiation alone. The 44.5°C treatment again abolished the TBE completely and led to more rapid tumour appearance, with latent periods remarkably similar to those seen after the consecutive treatments. Increasing the interval between treatments to 24 h produced little further change in the expression of stromal injury (data not shown). The lowest thermal dose appeared to produce a slight (but not significant) increase in latent periods.

### Discussion

The time of expression of radiation injury is dependent on both the size of the X-ray dose and the intermitotic time of the cells (Denekamp, 1986). Thus, damage to a slowly proliferating tissue such as the vasculature may not be expressed for many months after treatment, unless a stimulus is applied to force the cells into proliferation. In the tumour bed effect assay, the tumour cells provide this angiogenic stimulus and the reduced rate of tumour growth presumably reflects an inability of the vasculature to support the needs of the growing tumour as damaged endothelial cells continue to die and be removed. If tumour implantation is delayed the radiation damage remains latent (see Figure 3).

In contrast to the effects of radiation, thermal cell death occurs rapidly, showing no dependency on either dose or cell cycle time (Morris *et al.*, 1977; Law *et al.*, 1978). Therefore, when heat is combined with radiation, the time course of expression of damage will depend on whether a thermal sensitization of radiation effects or direct thermal cytotoxicity is produced.

In the present study a distinct difference was seen in the response of the stroma to X-rays and to heat. Using the growth of the Ca NT tumour as a measure of damage to the stroma, radiation induced both a marked dose-dependent increase in latency and a slight slowing of tumour growth. The dominant latency effects suggest that neovascularization in this tumour may occur very early after implantation, so that most of the radiation injury to the endothelial cells is expressed during the very early attempts at tumour growth. Since cells will continue to die at subsequent divisions, a protracted growth slowing effect is also observed. Only after the highest X-ray dose tested (20 Gy) and the longest latent period, does this slowing of growth of established tumours disappear. It appears likely that after this larger dose of radiation, *all* of the damage may be expressed during the early phase of growth. The restoration of a functional vascular network during the 60 days of latency would account for the subsequent increase in the rate of tumour growth. Recovery of the vascular bed must depend either on the proliferation of radiation survivors or the ingrowth of untreated vessels from the edge of the irradiated field.

A totally unexpected effect of hyperthermia was the earlier appearance of tumours in sites exposed to large heat doses. All fields preheated to 44.5°C, whether irradiated or not, gave rise to 2 mm tumours by 14–20 days, compared with 25 days in previously untreated sites. A smaller effect, but tending in the same direction, was seen in the sites heated with 43°C alone or at 4 or 24 hours after irradiation. It seems likely that these heat treatments produced enough generalised tissue damage to act as a stimulus for neovascularisation of the tissue to repair the mild thermal burn. Indeed the skin at the site of implant was already thickened when the tumour cells were inoculated after these severe treatments and moist breakdown and necrosis followed within days. The tumours arose in these sites before healing was complete. When a 4 week interval elapsed between heating and implant, by which time the implant site appeared normal, the growth of the tumours was no longer accelerated, but was closely similar to that in control sites (Figure 3).

Labelling studies of endothelial cells have shown that the angiogenic stimulus of an implanted tumour is not the most

**Figure 4** Dose-response curves for tumour latency after implantation into sites treated with X-rays alone (hatched area = mean ± 1 s.e.) or X-rays *immediately* followed by 1 h of s perthermia at the indicated temperatures (solid lines). Each point is the mean ± 1 s.e. for 6 treated mice.

effective stimulator of endothelial cell proliferation. Even faster proliferation has been observed in the capillaries infiltrating a subcutaneously implanted sponge (a model of a healing wound) (Hobson & Denekamp, unpublished) as well as in the vessels of the placenta during early pregnancy (Hobson & Denekamp, 1984). It is therefore suggested that the immediate cell death produced by the thermal treatment may elicit a similar angiogenic response to that induced by a mechanically inflicted wound. The addition of this 'wound healing' stimulus to the separate angiogenic stimulus of the implanted tumour cells might therefore result in a great increase in the rate of vascular proliferation, leading to an increased rate of tumour growth. An alternative explanation for the early appearance of tumours is that cellular damage to the tumour bed might prevent loss of cells from the inoculation site. Increased survival and growth of tumour cells injected into actively growing or inflamed tissues has previously been reported (van den Brenk *et al.*, 1974). However a continued enhancement of vascular proliferation throughout the healing of the thermal wound might better explain the prolonged increase in the rate of tumour growth.

The influence of hyperthermia on the radiation induced TBE was highly dependent on temperature. It is summarized in Figure 6. Only with 41.5°C immediately after X-rays was any significant enhancement of radiation damage seen. This was lost with a 4 or 24 h interval. Likewise with 43°C a reduction of the TBE was seen when a 4 or 24 h gap was allowed. The lack of effect seen with consecutive treatments perhaps reflects a balance of radiosensitization and cytotoxicity at this temperature. The 44.5°C treatment produced a response consistent with direct cytotoxicity alone, at all intervals it abolished the TBE, eliminated the dose dependence and accelerated the appearance of tumours.

The data we have presented contradict the previously published findings that there is no hyperthermia-induced TBE. Wheldon and Hingston (1982) used treatments of up to 60 min at 43.5°C which may have been too low to produce a significant change in the rate of tumour growth although the authors postulated that rapid recovery from thermal damage might occur before demands for neovascularization were made. Urano and Cunningham (1980) however used a heat dose of 43.5°C for 120 min, which should be equivalent in effectiveness to our own highest treatment dose of 44.5°C for 60 min if a 1°C change in temperature is assumed to be equivalent to a factor of 2 in heating time (Field, 1978). Although a growth rate reduction was observed when their fibrosarcoma cells were implanted into a previously irradiated mouse foot, no such changes were seen with hyperthermia (Urano & Cunningham, 1980).

Recently, experiments have been reported using a tumour system similar to the one used in the present study, i.e. stromal damage is expressed as changes in both latency and growth rate (Wondergem *et al.*, 1986). In their study an

Figure 5 Dose-response curves for tumour latency after implantation into sites treated with X-rays alone (hatched area) or X-rays followed *4 h later* by 1 h of hyperthermia (solid lines).

**Figure 6** The temperature dependence of the TBE, expressed as the tumour latency induced by hyperthermia applied either alone or at different intervals after a single dose of 16 Gy X-rays. The hatched region represents the latency (mean $\pm$ 1 s.e.) induced by 16 Gy X-rays alone.

increasing TBE was measured with increasing exposure to a fixed temperature when tumour implantation immediately followed a treatment of 15 to 60 min at 44°C. They did not, however, observe the earlier tumour appearance and faster rate of growth which we have reported following a severe thermal treatment, possibly the 0.5°C difference in temperature could account for this.

Wondergem *et al.* (1986) also investigated the response of the stroma to combined heat and radiation and, in agreement with the present study, measured a decreasing TBE with increasing heat dose and suggested that radiation damage to the stroma may be repaired during the recovery from thermal damage.

That stromal injury can influence the response of tumours to *in situ* radiation therapy is evidenced by the growth rate reduction frequently observed during tumour regrowth. Consequently, as suggested by Begg (1980), any agent having a different dose dependency for tumour cells and stromal cells could lead to an erroneous interpretation of regrowth delay curves; hyperthermia appears to fit into this category. The stromal radiosensitization measured with low doses of heat (this study and Wondergem *et al.*, 1986) may contribute to any tumour sensitization measured by *in situ* assays. Conversely, the reduction in the TBE produced by higher temperatures or longer times of treatment would result in a much *lower* estimate of treatment effectiveness of the combined modalities if measured by regrowth delay rather than local tumour control. Vascular damage has been shown to occur at lower temperatures in tumours (proliferating vasculature) than in normal tissue (quiescent vasculature) (Dudar and Jain, 1984; Dewhirst *et al.*, 1985). Thus, a particularly worrying consideration is the possibility that therapeutic treatment temperatures may induce enough stromal damage to actually stimulate vascular proliferation and thus rescue the surviving tumour cells. Our own data indicate that tumours regrowing after hyperthermia therapy *in situ* frequently do show an increased rate of growth (Hill and Smith, unpublished data). Similarly, a shorter time to relapse has been reported for canine tumours treated with combined heat and radiation, compared to radiation alone (Dewhirst *et al.*, 1983). Walker *et al.* (1982) also showed that in groups of tumours in which equal fractions had been cured with X-rays or heat, the recurrent tumours appeared much earlier after heat than after irradiation.

It is already recognised that tumour regression after hyperthermia is much faster than after radiation. This may have led to some of the current wave of clinical optimism for this modality. It is also recognised that vascular damage plays a significant part in the cell kill that leads to the regression. The present study shows that severe heat treatments, which in themselves produce normal tissue damage, may actually aid tumour repopulation by providing an additional stimulus to angiogenesis. Of course, for an *in situ* treatment, the higher heat dose would also be more cytotoxic to the tumour cells, and it is difficult to predict what balance would be achieved. It is clear, however, that another aspect of the vascular response needs to be considered both for hyperthermia alone and in combination with radiation.

We should like to thank Mr P. Russell and the animal house staff for their care in the production and maintenance of the mice, Mrs E. Marriott and Mrs J. Wilson for secretarial assistance, Prof. J.F. Fowler for constructive criticism and the Cancer Research Campaign for financial support.

### References

BARENDSEN, G.W. & BROERSE, J.J. (1969). Experimental radiotherapy of a rat rhabdomyosarcoma with 15 MeV neutrons and 300 kV X-rays. I. Effects of single exposures. *Eur. J. Cancer*, **5**, 373.

BEGG, A.C. (1980). Analysis of growth delay data: Potential pitfalls. *Br. J. Cancer*, **41**, Suppl. IV 93.

CLIFTON, K.H. & JIRTLE, R. (1975). Mammary carcinoma cell population growth in preirradiated and unirradiated transplant sites. *Radiology*, **117**, 459.

DENEKAMP, J. (1986). Cell kinetics and radiation biology. *Int. J. Radiat. Biol.*, **49**, 357.

DEWHIRST, M.W., SIM, D.A., GROSS, G. & KUNDRAT, M.A. (1985). Effect of heating rate on tumour and normal tissue microcirculatory function. In *Hyperthermic Oncology 1984*, Overgaard, J. (ed) **1**, p. 177. Taylor & Francis: London and Philadelphia.

DEWHIRST, M.W., SIM, D.A., WILSON, S., DE YOUNG, D. & PARSELLS, J.L. (1983). Correlation between initial and long-term responses of spontaneous pet animal tumors to heat and radiation or radiation alone. *Cancer Res.*, **43**, 5735.

DUDAR, T.E. & JAIN, J.T. (1984). Differential response of normal and tumor microcirculation to hyperthermia. *Cancer Res.*, **44**, 605.

EMAMI, B. & SONG, C.W. (1984). Physiological mechanisms in hyperthermia: A review. *Int. J. Radiat. Oncol. Biol. Phys.*, **10**, 289.

FAJARDO, L.F., EGBERT, B., MARMOR, J. & HAHN, G.M. (1980). Effects of hyperthermia in a malignant tumor. *Cancer*, **45**, 613.

FAJARDO, L.F., SCHREIBER, A.B., KELLY, N.I. & HAHN, G.M. (1985). Thermal sensitivity of endothelial cells. *Radiat. Res.*, **103**, 276.

FIELD, S.B. (1978). The response of normal tissues to hyperthermia alone or in combination with X-rays. In *Cancer Therapy by Hyperthermia and Radiation*, Streffer (ed) p. 37. Urban & Schwatzenberg: Baltimore and Munich.

FRANKL, O. & KIMBALL, C.P. (1914). Uber die Beeinflussing von Mäuse-Tumoren durch Röntgenstrahlen. *Wiener Klinische Wochenschrift*, **27**, 1448.

HEWITT, H.B. & BLAKE, E. (1968). The growth of transplanted murine tumours in pre-irradiated sites. *Br. J. Cancer*, **22**, 808.

HOBSON, B. & DENEKAMP, J. (1984). Endothelial proliferation in tumours and normal tissues: Continuous labelling studies. *Br. J. Cancer*, **49**, 405.

ITO, H., BARKLEY, T., PETERS, L.J. & MILAS, L. (1985). Modification of tumour response to cyclophosphamide and irradiation by pre-irradiation of the tumour bed: Prolonged growth delay but reduced curability. *Int. J. Radiat. Oncol. Biol. Phys.*, **11**, 547.

LAW, M.P., AHIER, R.G. & FIELD, S.B. (1978). The response of the mouse ear to heat applied alone or combined with X-rays. *Br. J. Radiol.*, **51**, 132.

MARMOR, J.B., HILERIO, F.J. & HAHN, G.M. (1979). Tumour eradication and cell survival after localized hyperthermia induced by ultrasound. *Cancer Res.*, **39**, 2166.

MORRIS, C.C., MYERS, R. & FIELD, S.B. (1977). The response of the rat tail to hyperthermia. *Br. J. Radiol.*, **50**, 576.

REINHOLD, H.S. and ENDRICH, B. (1986). Tumour microcirculation as a target for hyperthermia. *Int. J. Hyperthermia*, **2**, 111.

REINHOLD, H.S., WIKE-HOOLEY, J.L., VAN DEN BERG, A.P. & VAN DEN BERG-BLOK, A. (1985). Environmental factors, blood flow and microcirculation. In *Hyperthermic Oncology 1984*, Overgaard, J. (ed) **2**, p. 41. Taylor & Francis: London and Philadelphia.

ROFSTAD, E.K. & BRUSTAD, T. (1986). Primary and secondary cell death in human melanoma xenografts following hyperthermic treatment. *Cancer Res.*, **46**, 355.

SHELDON, P.W. & HILL, S.A. (1977). Hypoxic cell sensitizers and local control by X-ray of a transplanted tumour in mice. *Br. J. Cancer*, **35**, 795.

SONG, C.W. (1984). Effect of local hyperthermia on blood flow and microenvironment: A review. *Cancer Res.*, **44**, 4721.

SONG, C.W., KANG, M.S., RHEE, J.G. & LEVITT, S.H. (1980). Vascular damage and delayed cell death in tumours after hyperthermia. *Br. J. Cancer*, **41**, 309.

STENSTROM, K.W., VERMUND, H., MOSSER, D.G. & MARVIN, J.F. (1955). Effects of roentgen irradiation on the tumour bed. I. The inhibiting action of local pretransplantation roentgen irradiation (1500r) on the growth of mouse mammary carcinoma. *Radiat. Res.*, **2**, 180.

THOMLINSON, R.H. & CRADDOCK, R.A. (1967). The gross response of an experimental tumour to single doses of X-rays. *Br. J. Cancer*, **21**, 108.

URANO, M. & CUNNINGHAM, M. (1980). Insignificant tumor bed effect after pretransplantation hyperthermia. *Cancer Res.*, **40**, 26.

VAN DEN BRENK, H.A.S., STONE, M., KELLY, H., ORTON, C. & SHARPINGTON, C. (1974). Promotion of growth of tumour cells in acutely inflamed tissues. *Br. J. Cancer*, **30**, 246.

WALKER, A., WHELDON, T.E., BRUNTON, G.F. & ABDELAAL, A.S. (1982). Contrasting regrowth delay responses of a murine tumour to isocurative hyperthermia or X irradiation. *Br. J. Radiol.*, **55**, 780.

WHELDON, T.E. & HINGSTON, E.C. (1982). Differential effect of hyperthermia and X-irradiation on regrowth rate and tumour-bed effect for a rat sarcoma. *Br. J. Cancer*, **45**, 265.

WONDERGEM, J., BEGG, A.C. & HAVEMAN, J. (1986). Effects of hyperthermia and X-irradiation on mouse stromal tissue. *Int. J. Radiat. Biol.*, **50**, 65.


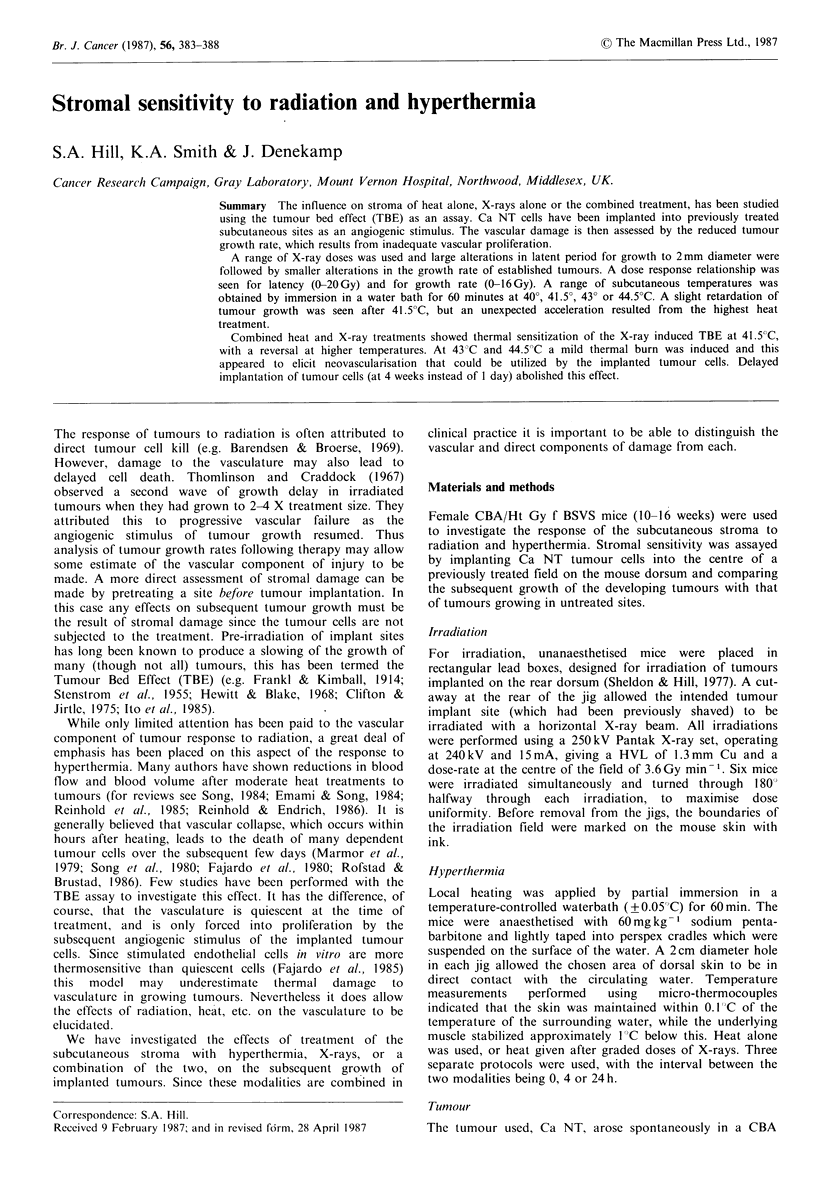

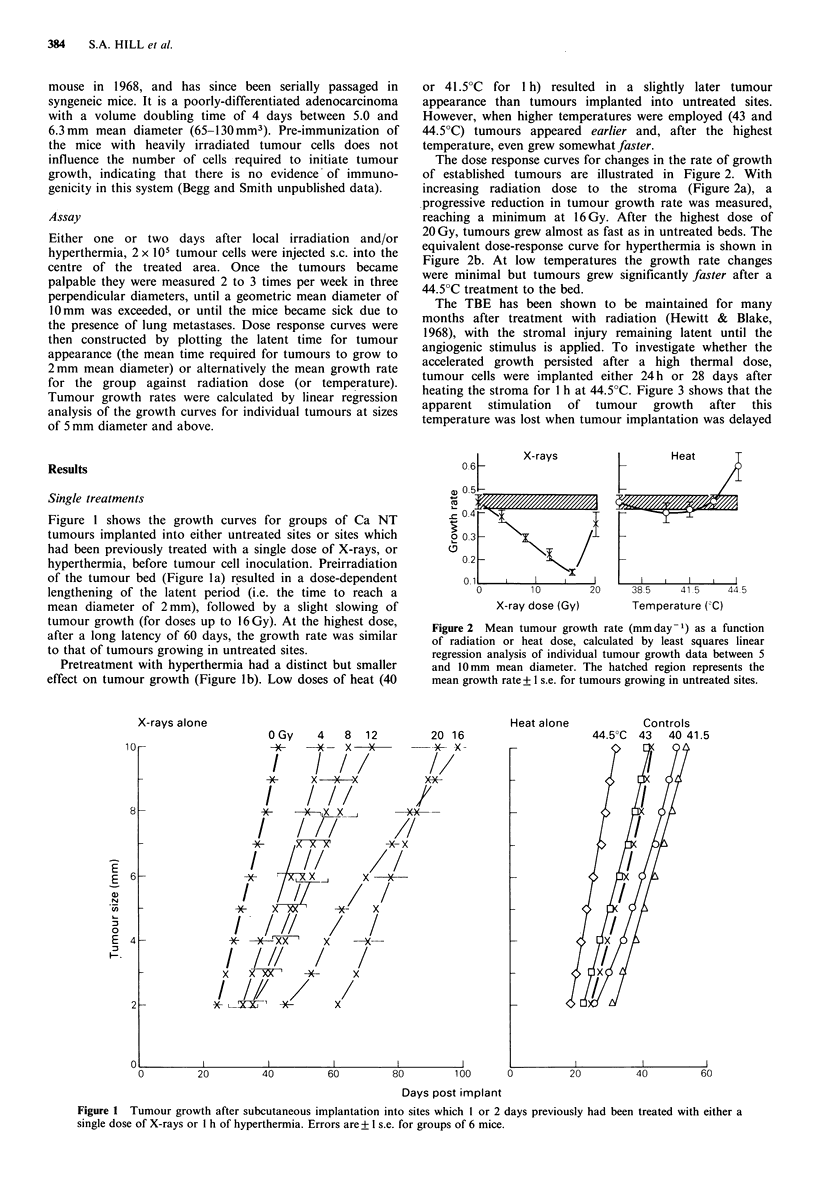

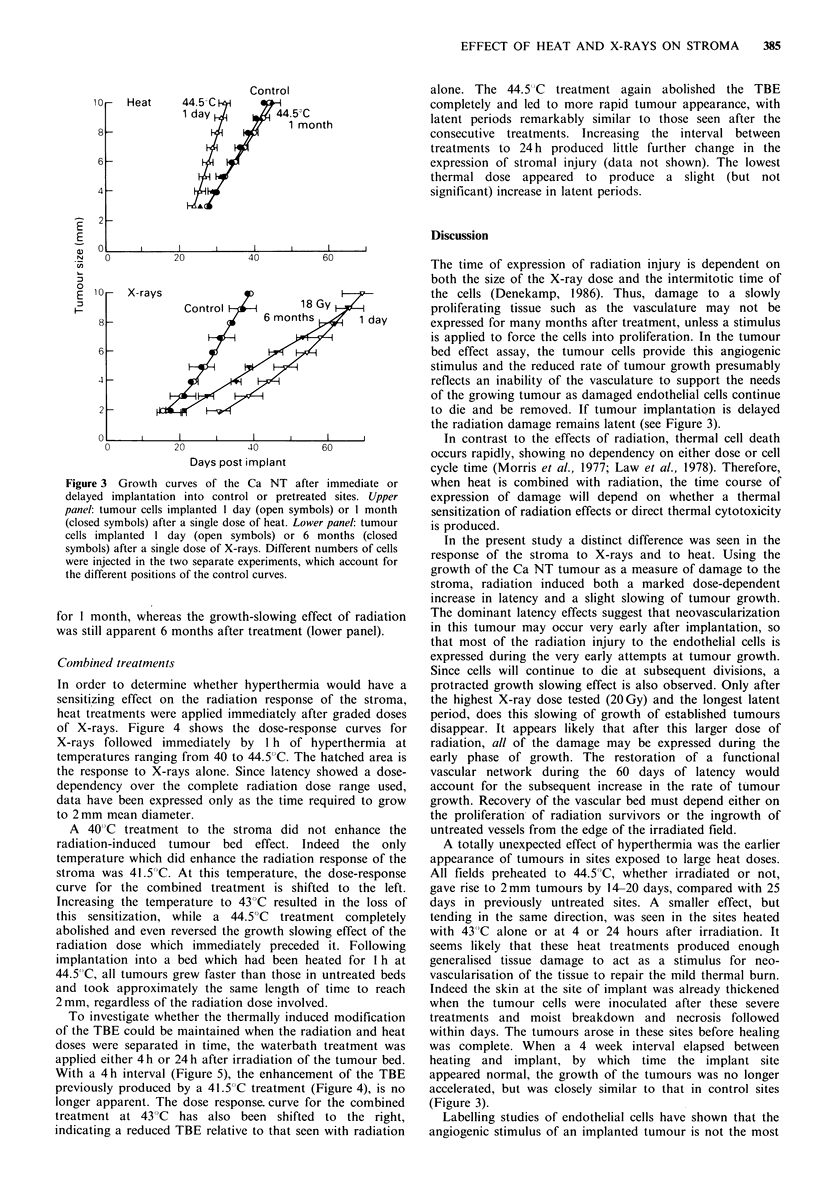

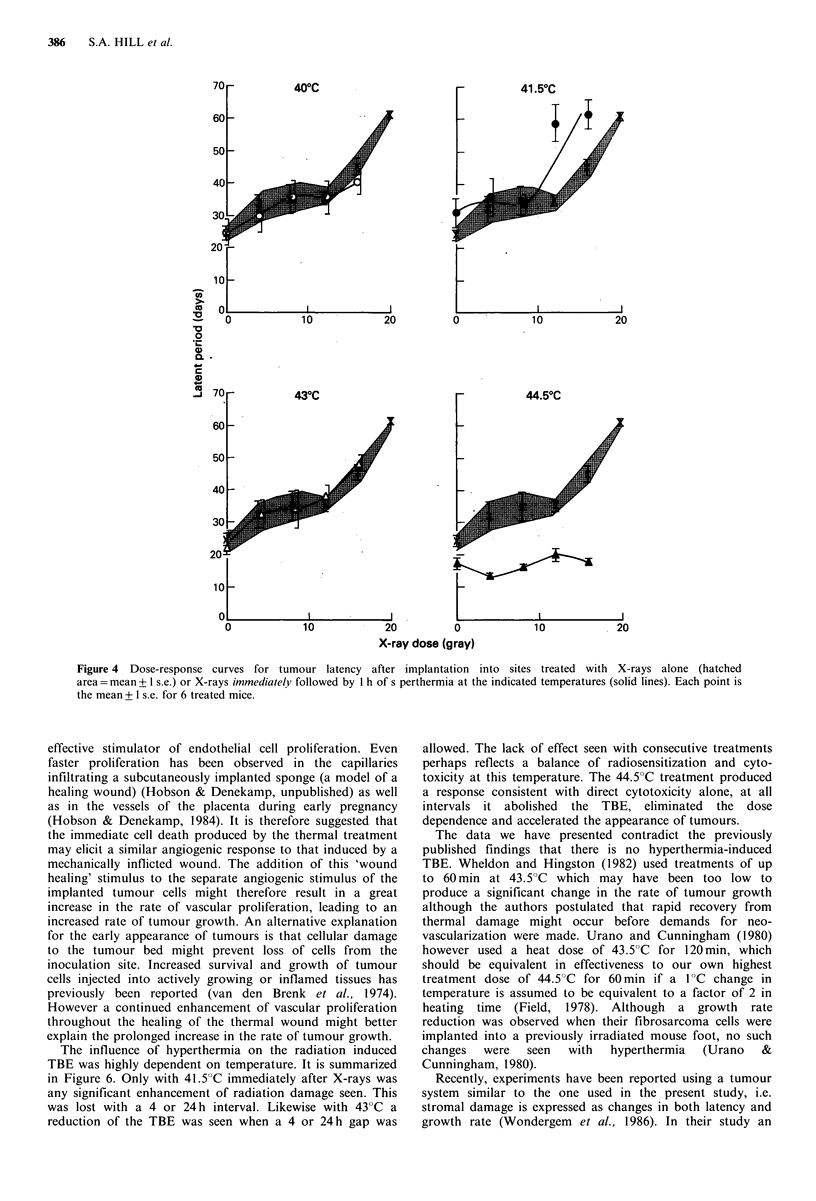

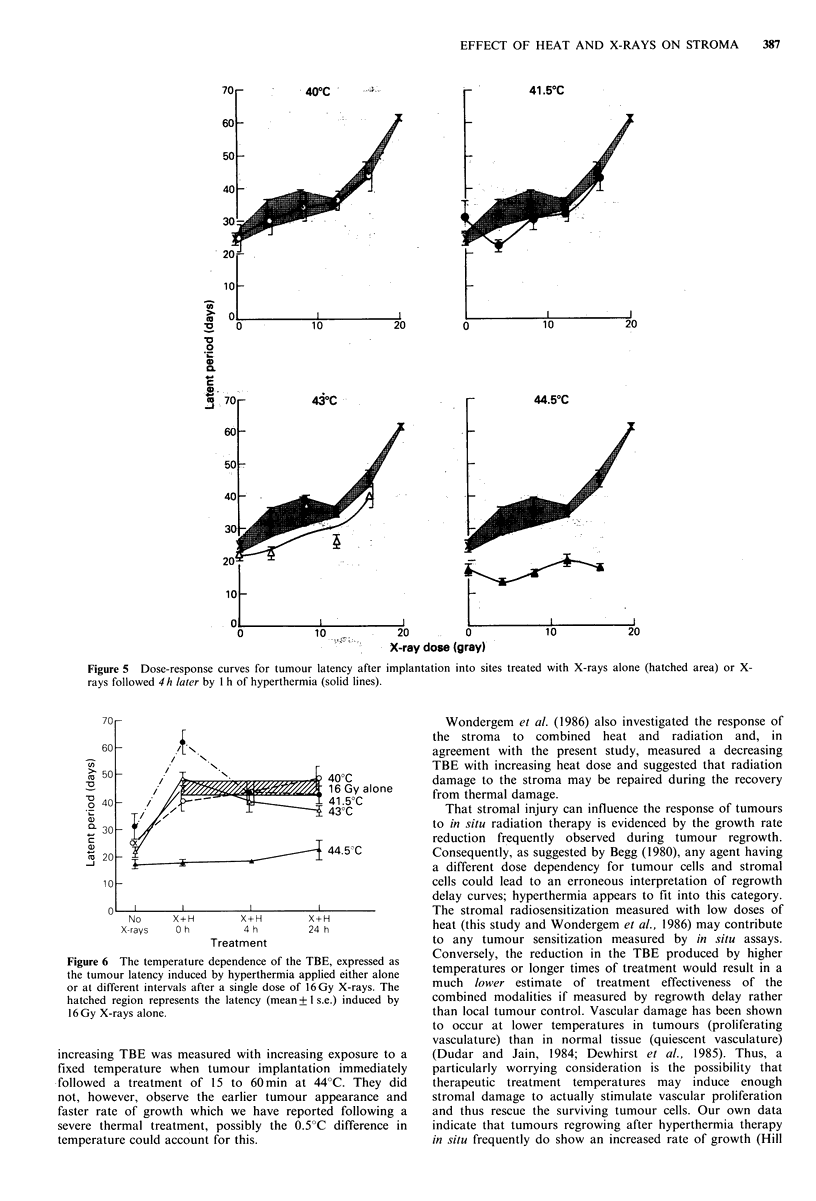

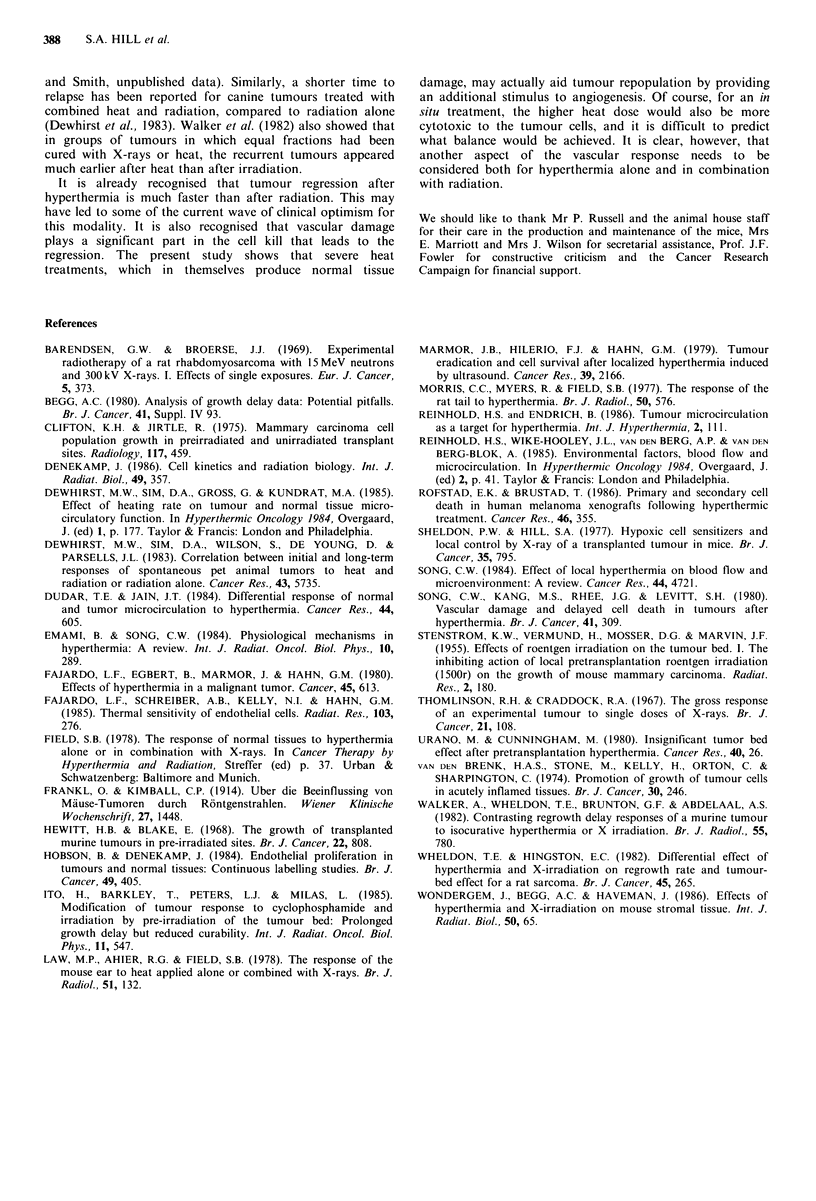


## References

[OCR_00729] Barendsen G. W., Broerse J. J. (1969). Experimental radiotherapy of a rat rhabdomyosarcoma with 15 MeV neutrons and 300 kV x-rays. I. Effects of single exposures.. Eur J Cancer.

[OCR_00739] Clifton K. H., Jirtle R. (1975). Mammary carcinoma cell population growth in preirradiated and unirradiated transplant sites. Viable tumor growth, vascularity, and the tumor-bed effect.. Radiology.

[OCR_00744] Denekamp J. (1986). Cell kinetics and radiation biology.. Int J Radiat Biol Relat Stud Phys Chem Med.

[OCR_00754] Dewhirst M. W., Sim D. A., Wilson S., DeYoung D., Parsells J. L. (1983). Correlation between initial and long-term responses of spontaneous pet animal tumors to heat and radiation or radiation alone.. Cancer Res.

[OCR_00760] Dudar T. E., Jain R. K. (1984). Differential response of normal and tumor microcirculation to hyperthermia.. Cancer Res.

[OCR_00765] Emami B., Song C. W. (1984). Physiological mechanisms in hyperthermia: a review.. Int J Radiat Oncol Biol Phys.

[OCR_00770] Fajardo L. F., Egbert B., Marmor J., Hahn G. M. (1980). Effects of hyperthermia in a malignant tumor.. Cancer.

[OCR_00774] Fajardo L. F., Schreiber A. B., Kelly N. I., Hahn G. M. (1985). Thermal sensitivity of endothelial cells.. Radiat Res.

[OCR_00790] Hewitt H. B., Blake E. R. (1968). The growth of transplanted murine tumours in pre-irradiated sites.. Br J Cancer.

[OCR_00794] Hobson B., Denekamp J. (1984). Endothelial proliferation in tumours and normal tissues: continuous labelling studies.. Br J Cancer.

[OCR_00799] Ito H., Barkley T., Peters L. J., Milas L. (1985). Modification of tumor response to cyclophosphamide and irradiation by preirradiation of the tumor bed: prolonged growth delay but reduced curability.. Int J Radiat Oncol Biol Phys.

[OCR_00806] Law M. P., Ahier R. G., Field S. B. (1978). The response of the mouse ear to heat applied alone or combined with X rays.. Br J Radiol.

[OCR_00811] Marmor J. B., Hilerio F. J., Hahn G. M. (1979). Tumor eradication and cell survival after localized hyperthermia induced by ultrasound.. Cancer Res.

[OCR_00816] Morris C. C., Myers R., Field S. B. (1977). The response of the rat tail to hyperthermia.. Br J Radiol.

[OCR_00820] Reinhold H. S., Endrich B. (1986). Tumour microcirculation as a target for hyperthermia.. Int J Hyperthermia.

[OCR_00830] Rofstad E. K., Brustad T. (1986). Primary and secondary cell death in human melanoma xenografts following hyperthermic treatment.. Cancer Res.

[OCR_00849] STENSTROM K. W., VERMUND H., MOSSER D. G., MARVIN J. F. (1955). Effects of roentgen irradiation on the tumor bed. I. The inhibiting action of local pretransplantation roentgen irradiation (1500 r alpha) on the growth of mouse mammary carcinoma.. Radiat Res.

[OCR_00835] Sheldon P. W., Hill S. A. (1977). Hypoxic cell radiosensitizers and local control by X-ray of a transplanted tumour in mice.. Br J Cancer.

[OCR_00844] Song C. W., Kang M. S., Rhee J. G., Levitt S. H. (1980). Vascular damage and delayed cell death in tumours after hyperthermia.. Br J Cancer.

[OCR_00856] Thomlinson R. H., Craddock E. A. (1967). The gross response of an experimental tumour to single doses of x-rays.. Br J Cancer.

[OCR_00861] Urano M., Cunningham M. (1980). Insignificant tumor bed effect after pretransplantation hyperthermia.. Cancer Res.

[OCR_00865] Van Den Brenk H. A., Stone M., Kelly H., Orton C., Sharpington C. (1974). Promotion of growth of tumour cells in acutely inflamed tissues.. Br J Cancer.

[OCR_00870] Walker A., Wheldon T. E., Brunton G. F., Abdelaal A. S. (1982). Contrasting regrowth delay responses of a murine tumour to isocurative hyperthermia or X irradiation.. Br J Radiol.

[OCR_00876] Wheldon T. E., Hingston E. C. (1982). Differential effect of hyperthermia and x-irradiation on regrowth rate and tumour-bed effect for a rat sarcoma.. Br J Cancer.

[OCR_00881] Wondergem J., Begg A. C., Haveman J. (1986). Effects of hyperthermia and X-irradiation on mouse stromal tissue.. Int J Radiat Biol Relat Stud Phys Chem Med.

